# Graph theory analysis of cortical thickness networks in adolescents with d‐transposition of the great arteries

**DOI:** 10.1002/brb3.834

**Published:** 2018-01-18

**Authors:** Christopher G. Watson, Christian Stopp, Jane W. Newburger, Michael J. Rivkin

**Affiliations:** ^1^ Graduate Program for Neuroscience Boston University Boston MA USA; ^2^ Department of Neurology Boston Children's Hospital Boston MA USA; ^3^ Department of Cardiology Boston Children's Hospital Boston MA USA; ^4^ Department of Pediatrics Harvard Medical School Boston MA USA; ^5^ Department of Radiology Boston Children's Hospital Boston MA USA; ^6^ Department of Psychiatry Boston Children's Hospital Boston MA USA; ^7^ Department of Neurology Harvard Medical School Boston MA USA

**Keywords:** Congenital heart disease, cortical thickness, d‐TGA, graph theory, MRI

## Abstract

**Objective:**

Adolescents with d‐transposition of the great arteries (d‐TGA) who had the arterial switch operation in infancy have been found to have structural brain differences compared to healthy controls. We used cortical thickness measurements obtained from structural brain MRI to determine group differences in global brain organization using a graph theoretical approach.

**Methods:**

Ninety‐two d‐TGA subjects and 49 controls were scanned using one of two identical 1.5‐Tesla MRI systems. Mean cortical thickness was obtained from 34 regions per hemisphere using Freesurfer. A linear model was used for each brain region to adjust for subject age, sex, and scanning location. Structural connectivity for each group was inferred based on the presence of high inter‐regional correlations of the linear model residuals, and binary connectivity matrices were created by thresholding over a range of correlation values for each group. Graph theory analysis was performed using packages in R. Permutation tests were performed to determine significance of between‐group differences in global network measures.

**Results:**

Within‐group connectivity patterns were qualitatively different between groups. At lower network densities, controls had significantly more long‐range connections. The location and number of hub regions differed between groups: controls had a greater number of hubs at most network densities. The control network had a significant rightward asymmetry compared to the d‐TGA group at all network densities.

**Conclusions:**

Using graph theory analysis of cortical thickness correlations, we found differences in brain structural network organization among d‐TGA adolescents compared to controls. These may be related to the white matter and gray matter differences previously found in this cohort, and in turn may be related to the cognitive deficits this cohort presents.

## 1 INTRODUCTION

Congenital heart disease (CHD) is the most commonly occurring congenital anomaly (Tennant, Pearce, Bythell, & Rankin, 2010). Due to improvements in medical and surgical care, a steadily increasing proportion of those born with CHD are surviving into adolescence and adulthood. Research has increasingly focused on the behavioral and neuropsychological deficits present throughout development in this cohort (Bang et al., 2013; Bellinger et al., 2011; Cassidy, White, DeMaso, Newburger, & Bellinger, 2015; Heinrichs et al., 2014; Marino et al., 2012). Recently, evidence of differences in brain structure of adolescents with CHD has accumulated, and some of these differences have been associated with cognition (Rivkin et al., 2013; Rollins et al., 2014; M von Rhein et al., 2014).

D‐transposition of the great arteries (d‐TGA) is a form of CHD that is corrected by the arterial switch operation using cardiopulmonary bypass in early infancy. Brain abnormalities can be seen on MRI pre‐ and postoperatively, as well as in utero (Clouchoux et al., 2013; Licht et al., 2009; Limperopoulos et al., 2010; Ortinau, Beca, et al., 2012). Although white matter is most often affected, reduced cortical gray matter volume in the frontal and parietal lobes is also present several months after surgery (Ortinau, Beca, et al., 2012; Watanabe et al., 2009). Our group, using region‐of‐interest analyses, has shown that these measurable differences in brain structure have not returned to normal by adolescence in d‐TGA patients (Rivkin et al., 2013; Rollins et al., 2014; Watson et al., 2016). While this regional approach to studying brain anatomical differences is helpful, it does not take into account the global organization of the brain, that is, its network structure. In a separate analysis of a subset of the same d‐TGA adolescents, we have shown that differences exist in global brain organization based on white matter connectivity (Panigrahy et al., 2015).

Strong inter‐regional cortical thickness correlations have been established as measures of structural connectivity, as regions with high structural covariance may share a maturational trajectory due to direct axonal connections or to a mutual influence (Alexander‐Bloch, Raznahan, Bullmore, & Giedd, 2013). In healthy subjects, there is a moderate agreement between networks constructed from positive cortical thickness correlations and diffusion tensor imaging (DTI) tractography (Gong, He, Chen, & Evans, 2012). A developmental study of gray matter covariance networks showed consistency between structural networks and known functional connectivity networks (Zielinski, Gennatas, Zhou, & Seeley, 2010). Further, networks constructed from cortical thickness data possess the “small‐world” property and have distinct modules/communities of vertices, similar to network qualities derived from DTI tractography and resting‐state functional MRI data (Achard, Salvador, Whitcher, Suckling, & Bullmore, 2006; Chen, He, Rosa‐Neto, Germann, & Evans, 2008; Chen, Liu, Gross, & Beaulieu, 2013; He, Chen, & Evans, 2007; Iturria‐Medina, Sotero, Canales‐Rodríguez, Alemán‐Gómez, & Melie‐García, 2008). These properties are present as early as 1 month of age and persist throughout development (Fan et al., 2011; Khundrakpam et al., 2013). Finally, cortical thickness networks have been used to elucidate organizational brain differences in patients with Alzheimer's disease, Parkinson's disease, and epilepsy (Bernhardt, Chen, He, Evans, & Bernasconi, 2011; He, Chen, & Evans, 2008; Pereira et al., 2015).

To the best of our knowledge, analysis of gray matter connectivity has not been employed to discern related organizational networks in CHD patients of any age and may contribute to a more complete picture of the structural brain differences in this cohort. Here, we use a graph theoretical approach to analyze brain networks based on cortical thickness correlations to compare brain structure in a group of adolescents born with d‐TGA corrected surgically in early infancy with that of typically developing control adolescents.

## 2 METHODS

### 2.1 Subjects

Adolescents in the d‐TGA group were recruited from the Boston Circulatory Arrest Study, as previously described (Bellinger et al., 1995; Newburger et al., 1993). In brief, d‐TGA subjects underwent the arterial switch procedure before 3 months of age between April 1988 and February 1992 at Boston Children's Hospital (BCH) (Bellinger et al., 1995; Newburger et al., 1993). Exclusion criteria included: known risk factors for brain disorders (e.g., history of closed head injury with loss of consciousness), any contraindication to acquisition of MRI data (e.g., metal implants), Trisomy 21, and adolescents with forms of CHD other than d‐TGA requiring surgical correction. The criteria used to recruit healthy control subjects were adapted from those of the NIH MRI study of normal brain development (Almli, Rivkin, McKinstry, & Brain Development Cooperative Group, 2007; Evans & Brain Development Cooperative Group, 2006). This study was approved by the Institutional Review Board and adhered to institutional guidelines and the Declaration of Helsinki. Parents provided informed consent and adolescents provided assent.

### 2.2 MRI acquisition

Subjects were scanned on identical GE Twinspeed 1.5 Tesla (T) systems (General Electric, Milwaukee, WI, USA) with a quadrature head coil at either BCH or Beth Israel Deaconess Medical Center (BIDMC). The volumetric series for each subject was acquired using a Spoiled Proton Gradient Recalled (SPGR) sequence with parameters: TR/TE = 35 ms/6 ms, flip angle = 45 degrees, acquisition matrix = 256 × 256, FOV = 220 mm, slice thickness = 1.5 mm, with resultant voxel size = 0.86 × 0.86 × 1.5 mm^3^. The images were inspected by a radiologist to assure data quality and detect structural abnormalities (e.g., tumors, stroke, etc.).

### 2.3 Cortical thickness calculation

Images were processed using *Freesurfer v5.0* (A.A. Martinos Center for Biomedical Imaging, Massachusetts General Hospital). The technical details are described elsewhere (Dale, Fischl, & Sereno, 1999; Fischl & Dale, 2000; Fischl, Sereno, & Dale, 1999). Briefly, MRI images are first partitioned into white matter, gray matter, and cerebrospinal fluid. The outer pial surface of the brain is calculated, as is the surface comprising the white matter/gray matter junction. Cortical thickness is obtained by taking the distance between these two surfaces at every data point. Finally, the cortical surface is parcellated into distinct units based on gyral and sulcal anatomy (Fischl et al., 2004). The Desikan‐Killiany atlas, which contains 34 regions per hemisphere, was used for the parcellation (Desikan et al., 2006). Mean cortical thickness was obtained for all regions for each subject.

### 2.4 Network construction

All statistics were performed in *R v3.2* (R Core Team, 2017), using functions in the packages *igraph* and *brainGraph* (https://cran.r-project.org/web/packages/brainGraph) (Csardi & Nepusz, 2006; Kolaczyk & Csárdi, 2014). First, a general linear model was specified for each brain region, with mean cortical thickness as the outcome variable and age, sex, and scanner location (BCH or BIDMC) as covariates. Next, Pearson correlation coefficients between the model residuals for all pairs of regions were calculated, creating an adjacency matrix of size 68 × 68 for each group.

The adjacency matrix of each group was binarized by thresholding and removing any correlations lower than the threshold. Negative correlations were not considered, as these likely do not represent real anatomic connections in the brain (Gong et al., 2012). To ensure equal network sizes for both groups, the thresholds were chosen to result in a specific density (i.e., ratio of the number of edges present in the network to total possible number of edges); a range of densities from 0.05 to 0.40 (step size: 0.01) was investigated. Since correlations were generally larger in the control group, the correlation threshold for each specified density was correspondingly larger (i.e., an equal threshold for each group would have resulted in a higher density in the control network). The networks created from these matrices were un‐directed, un‐weighted, and simple (i.e., no loops).

### 2.5 Network metrics

Vertex‐ (i.e., region‐) and graph‐level metrics were calculated for both groups at each density. For visualization and group analysis purposes, a density of 22% was chosen, as this was the lowest density for which at least 95% of vertices were connected for both groups. This density is within the range used in several other studies (Bernhardt et al., 2011; He et al., 2008; Khundrakpam et al., 2013; Nie, Li, & Shen, 2013).

#### 2.5.1 Vertex importance

Vertex degree (the number of connections of a vertex), betweenness centrality (the number of shortest paths a vertex lies on), and nodal efficiency were used as measures of vertex importance. A vertex was considered to be a hub if its betweenness centrality was at least one standard deviation greater than the mean across all vertices for that density (Bernhardt et al., 2011; Hosseini et al., 2013; Tijms et al., 2013; Wang et al., 2013). Since regions classified as hubs may change across densities, we report those regions which were classified as hubs in at least half (i.e., 18/36) of the densities investigated. We also investigated rich‐club organization for each group (Baker et al., 2015; Colizza, Flammini, Serrano, & Vespignani, 2006; van den Heuvel & Sporns, 2011). A rich‐club is a group of vertices with high degree that are significantly more likely to be connected to each other compared to a set of equivalent random graphs. For both groups, we calculated the rich‐club coefficient, ϕ, for each degree (from 1 to the maximum degree present in the network). We normalized ϕ (denoted ϕ_norm_) by dividing by the average over a set of 1,000 equivalent random graphs (for each group and density). The random graphs were generated by randomly rewiring edges in the group‐specific graphs for 10,000 iterations, keeping constant the graph's density and degree sequence (Maslov & Sneppen, 2002). Rich‐club organization is considered present if ϕ_norm_ > 1 for a range of degree thresholds. To determine a degree boundary, we used the “rich‐core” algorithm of Ma and Mondragón (2015), which sorts the vertices from highest to lowest degree; the boundary is calculated as the local maximum of the function of degree rank and number of connections to higher‐degree vertices (Ma & Mondragón, 2015). For simplicity, we used the maximum boundary value across subject groups.

#### 2.5.2 Network segregation and integration

Network segregation was assessed with three metrics. Modularity measures the strength of a given network partition. Higher modularity indicates that vertices belonging to the same network module (or community) are more connected to each other than they are to vertices of a different module. The Louvain algorithm was used to partition the networks into communities and compute the modularity (Blondel, Guillaume, Lambiotte, & Lefebvre, 2008). Degree assortativity is a related metric that measures the strength with which vertices of similar degree connect to one another; higher assortativity indicates that high‐degree vertices are more likely to connect to other high‐degree vertices compared to low‐degree vertices. We also introduce lobe assortativity, which measures the number of inter‐lobar connections relative to intra‐lobar connections. This is equivalent to calculating the modularity of the network if it were a priori partitioned into the major lobes of the brain (i.e., frontal, parietal, temporal, occipital, insula, and cingulate). Higher values of lobe assortativity are present in networks with relatively fewer inter‐lobar connections.

Small‐worldness represents a balance between segregation and integration (Watts & Strogatz, 1998). Small world parameters clustering coefficient (*C*; the tendency of a vertex's neighbors to be connected to one another) and characteristic path length (*L*; the average of shortest path lengths between all vertices) were calculated, along with the average of each parameter from all random graphs (denoted *C*_rand_ and *L*_rand_, respectively) for each group (Humphries & Gurney, 2008; Watts & Strogatz, 1998). The small‐world index (σ) is the ratio of the normalized *C* to the normalized *L* (calculated as γ = *C*/*C*_rand_ and λ = *L*/*L*_rand_, respectively), and a network is considered to possess the “small world” property if σ > 1.

Since the networks in this study are generated from correlations, they will tend to have a higher‐than‐expected level of clustering (Hosseini & Kesler, 2013; Zalesky, Fornito, & Bullmore, 2012). As a result, the random graphs generated by a simple rewiring procedure may not be entirely appropriate, as they will have very low clustering by design (Newman, 2010). Thus, as an alternative we generated (for each density and each group) 100 random networks while controlling for global clustering using a Markov Chain process (Bansal, Khandelwal, & Meyers, 2009). We then calculated an alternate small‐world index, ω:(Telesford, Joyce, Hayasaka, Burdette, & Laurienti, 2011)

$$\omega =\frac{L_{\mathrm{rand}}}{L}-\frac{C}{C_{\mathrm{latt}}}$$

Here, *C*_latt_ is the mean clustering coefficient of a set of equivalent lattices; i.e., the graphs generated from the Markov Chain process in this case with *L*_rand_ as previously described. A network is considered a small‐world network if −0.5 ≤ ω ≤ 0.5; networks with ω closer to −1 are more similar to a lattice, and networks with ω closer to 1 are more similar to a random network. Networks with ω = 0 are considered to have a balance between global clustering and characteristic path length.

#### 2.5.3 Network closeness

Edge distances were calculated as the Euclidean distance in MNI coordinates (in mm) between centroids of pairs of connected regions (Alexander‐Bloch, Vértes, et al., 2013; Bassett et al., 2008; He et al., 2007). Vertex distances were calculated as the mean distance of all edges connecting a given vertex to all other vertices (Alexander‐Bloch, Vértes, et al., 2013). Similarly, characteristic path length (*L*) serves as a measure of the global closeness of a network.

#### 2.5.4 Asymmetry and hemispheric efficiency

A measure of asymmetry, the asymmetry index, was calculated as the difference in the number of left and right hemisphere intrahemispheric connections, divided by the average number of intrahemispheric connections of both hemispheres. An asymmetry index <0 indicates that the network has more intrahemispheric connections in the right compared to the left hemisphere. Additionally, we separated the group networks into isolated left and right hemisphere networks (Iturria‐Medina et al., 2011; Li et al., 2015). We then calculated global efficiency (the inverse of the edges' shortest path lengths, averaged over all edges) and local efficiency (the efficiency of a subnetwork comprising a vertex's neighbors, averaged across all vertices) of each hemisphere separately.

#### 2.5.5 Network robustness

Network robustness was assessed using “targeted attack” and “random failure” analyses, in addition to calculating global vulnerability (Albert, Jeong, & Barabási, 2000; Bernhardt et al., 2011; He et al., 2008; Iturria‐Medina et al., 2008; Romero‐Garcia, Atienza, Clemmensen, & Cantero, 2012; Wang et al., 2013). In a targeted attack analysis, vertices are sorted in descending order of betweenness centrality. The size of the largest connected component (the number of vertices that are reachable from any other vertex) is computed, and then the vertex with the highest betweenness is removed. After removal of that vertex and its connections, the size of the largest connected component is computed for this new network. These steps are repeated until all vertices have been removed. In a random failure analysis, vertices are removed in random order, and the size of the largest connected component is recorded after each removal. This was repeated 1,000 times and averaged over all iterations. Both targeted attack and random failure analyses were also performed with edge removals, using the same procedure except edges were sorted in decreasing order of edge betweenness. Vulnerability (*V*) is calculated across all network vertices; for vertex *i,*

$$V\left(i\right)=1-\frac{E_{\mathrm{glob}}\left(i\right)}{E_{\mathrm{glob}}}$$

where *E*_glob_(*i*) is the global efficiency of the network after removing vertex *i*. Global vulnerability is the maximum across all vertices; higher values indicate that the network is less stable in the presence of vertex removal.

### 2.6 Network analysis

Between‐group difference in the set of inter‐regional correlations was determined by a two‐sample *t*‐test. Permutation testing was performed to assess group differences in global network measures (i.e., number of hubs, modularity, assortativity, clustering coefficient, characteristic path length, edge asymmetry, global and local efficiency, and vulnerability) and vertex‐level measures (degree, betweenness centrality, and nodal efficiency). Each subject was randomly assigned to one of two groups (of the same size as the d‐TGA and control groups), and then we followed the procedure for network construction described above. This resulted in two networks for which we calculated the between‐group difference in the area under the curve (AUC) across densities. We calculated 5,000 permutations for both global and vertex measures. For single hemisphere analyses, we performed 1,000 permutations per density. Permutation *p* values were calculated as the proportion of times the randomized set of between‐group differences was greater than (for number of hubs, clustering coefficient, right hemisphere global and local efficiency, and vertex degree) or less than (for modularity, assortativity, characteristic path length, edge asymmetry, left hemisphere global and local efficiency, and vulnerability) the observed between‐group difference of control and d‐TGA subjects. Similarly, for the rich‐club analysis, *p* values were calculated as the proportion of times the rich‐club coefficient of the random graphs exceeded that of each group network for each degree threshold, and adjusted for false discovery rate (FDR) (Benjamini & Hochberg, 1995). Group differences in edge distance and in mean vertex distance of hub regions were assessed using a two‐sample Wilcoxon rank‐sum test at each density, with *p* values adjusted for FDR.

## 3 RESULTS

### 3.1 Subjects

Demographic and medical characteristics of the 92 d‐TGA subjects and 49 control subjects have been described previously and is shown in Table 1; of note, the d‐TGA subjects were significantly older and more likely to be male than control subjects (Watson et al., 2016).

**Table 1:** Demographic characteristics of d‐TGA and control subjects

Variables	d‐TGA (*n* ** **=** **92)	Control (*n* ** **=** **49)	*p*‐Value^a^
Age at MRI (y)	16.1 (15.8–16.4)	15.7 (14.2–16.3)	<.001
Sex (F)	22 (24)	29 (59)	<.001
Scanner (BCH)	68 (74)	34 (69)	.56
Race			
Asian	2 (1)	2 (4)	.006
Black	1 (1)	7 (14)	
Black/Asian	1 (1)	0 (0)	
White	86 (93)	39 (80)	
White/Asian	2 (1)	1 (1)	
Time to first surgery (d)	6 (4–9)	–	–

y, years; F, female; BCH, Boston Children's Hospital; d, days.

Values are *n* (%) or median (IQR).

a Determined by Fisher exact test for categorical variables and Wilcoxon test for continuous variables represented with medians.

Abnormality on structural MRI was more common in d‐TGA subjects. In the d‐TGA group, 33 (36%) had at least one finding: 7 (8%) subjects had evidence of prior infarction, 21 (23%) showed brain mineralization, 2 (2%) showed abnormal T2 hyperintensities, and 8 (9%) had minor malformations. Only 2 (4%) control subjects had evidence any structural abnormality (both minor malformations).

### 3.2 Cortical thickness covariance

The set of inter‐regional correlations of cortical thicknesses was significantly greater in the control group compared to the d‐TGA group (control mean: *r* = .23; d‐TGA mean: *r = *.19; *p* < .001). Across the range of network densities, the mean difference in correlation thresholds used for network construction was .057. The threshold resulting in a network density of 0.22 was 0.378 for the control group and 0.318 for the d‐TGA group.

### 3.3 Network hubs

**Figure 1:**
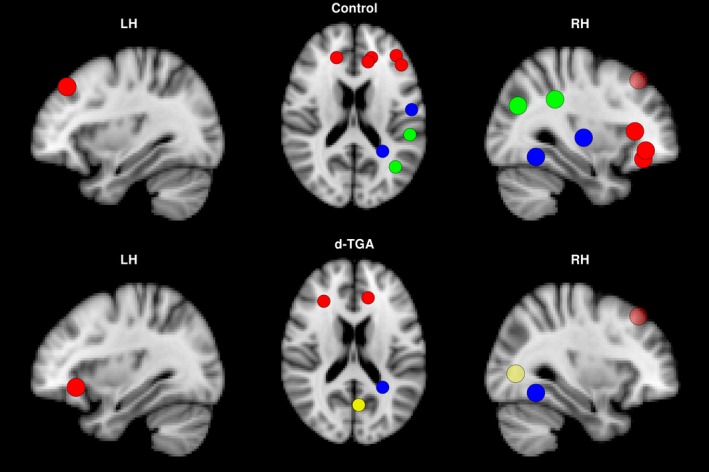
Hub regions in the control and d‐TGA groups. Hub regions are displayed for the control (top) and d‐TGA (bottom) groups. These regions were determined to be hubs in at least half of all densities tested. The left column depicts a sagittal view of the left hemisphere, the center column depicts an axial view, and the right column depicts a sagittal view of the right hemisphere. Colors of individual vertices are based on membership in brain lobes — red: frontal; green: parietal; blue: temporal; yellow: occipital

The distribution of hubs differed both quantitatively and qualitatively between groups. Regions determined to be hubs in at least half of all densities are shown for both groups in Figure 1, overlaid onto the brain. There were 9 hubs in the control group and 4 hubs in the d‐TGA group (Table 2, top and bottom, respectively). Hubs in both groups tended to be in the right hemisphere (8/9 control; 3/4 d‐TGA). Only the right superior frontal gyrus and right fusiform gyrus were common to both groups. The d‐TGA group rarely demonstrated more hubs than the control group across densities; however, the between‐group difference in AUC for the number of hubs was not significant (*p*_AUC_ = .08). Furthermore, there were no significant between‐group differences in any measures of vertex importance (degree, betweenness centrality, and nodal efficiency).

**Table 2:** Hub region locations

Region	Hemisphere	Lobe
Control		
Superior frontal gyrus	L	Frontal
Lateral orbitofrontal cortex	R	Frontal
Pars triangularis	R	Frontal
**Superior frontal gyrus**	R	Frontal
Superior temporal gyrus	R	Temporal
**Fusiform gyrus**	R	Temporal
Supramarginal gyrus	R	Parietal
Inferior parietal lobule	R	Parietal
Pars orbitalis	R	Frontal
d‐TGA		
Lateral orbitofrontal cortex	L	Frontal
**Superior frontal gyrus**	R	Frontal
**Fusiform gyrus**	R	Temporal
Lingual gyrus	R	Occipital

L, left; R, right.

Regions in bold typeface are hub regions in both the control and d‐TGA groups for at least half of all densities tested.

### 3.4 Rich‐club organization

**Figure 2:**
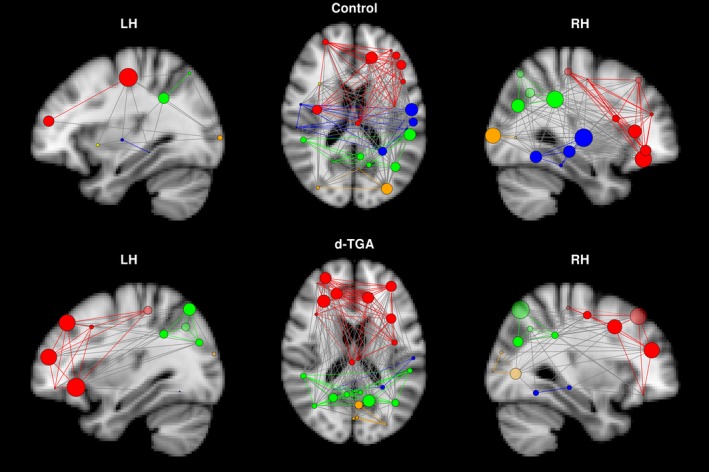
Rich‐club regions in the control and d‐TGA groups at a density of 0.22. Rich‐club regions and their connections are displayed for the control (top) and d‐TGA (bottom) groups. The left column depicts a sagittal view of the left hemisphere, the center column depicts an axial view, and the right column depicts a sagittal view of the right hemisphere. Colors of individual vertices are based on membership in brain lobes — red: frontal; green: parietal; blue: temporal; orange: occipital; yellow: insula

Rich‐club organization was present in both groups for a range of degree thresholds. The “rich‐core” degree boundary was *k* = 19 for the control group (containing 26 vertices, or 38% of the total network) and *k* = 16 for the d‐TGA group (containing 27 vertices, or 40% of the total network). When thresholding by the maximum across groups (i.e., *k* = 19), as in the distribution of hub regions, rich‐club regions were predominantly right‐hemispheric in the control group (Figure 2, top). However, the distribution of rich‐club regions in the d‐TGA group was relatively symmetric across hemispheres (Figure 2, bottom). Based on permutation analysis, vertex degree was significantly greater in the control group compared to the d‐TGA group in right pars triangularis and right superior temporal gyrus (*p* = .02 and *p* = .002 uncorrected, respectively).

### 3.5 Network segregation

**Figure 3:**
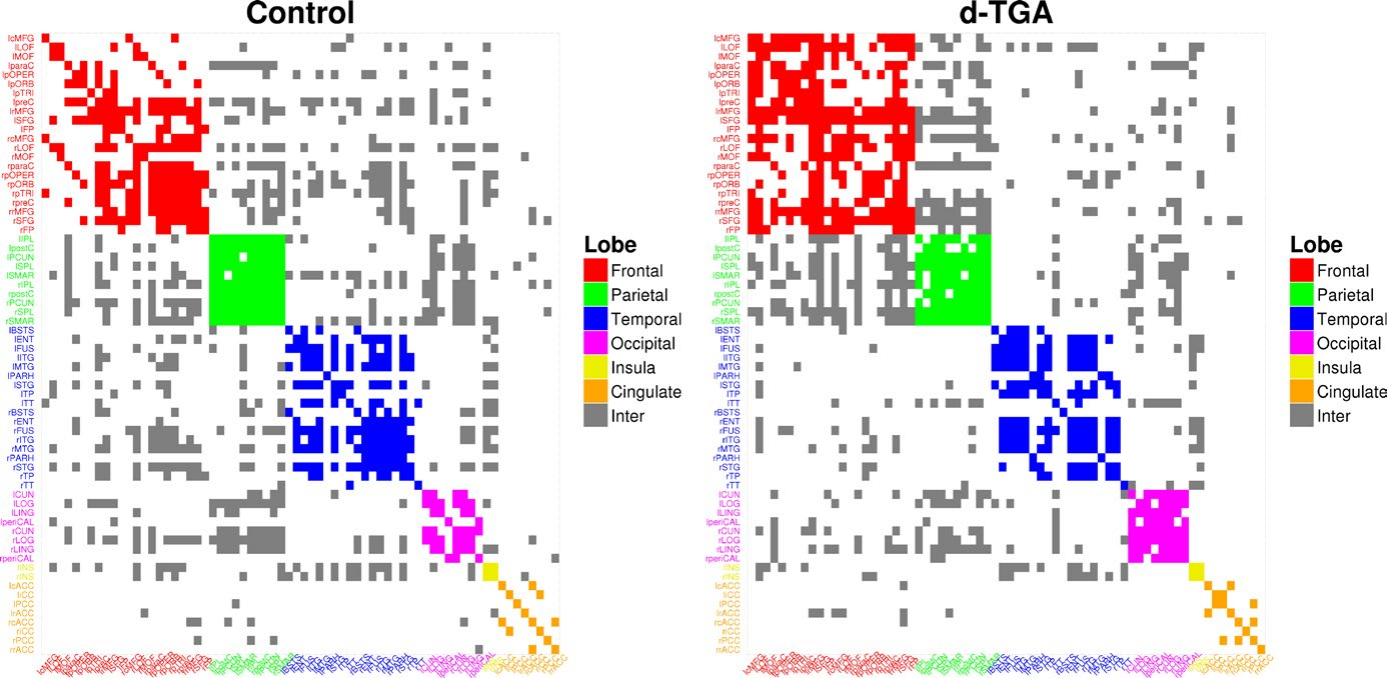
Adjacency matrix plots for the control and d‐TGA groups at a density of 0.22. Colors of individual vertices are based on membership in brain lobes — red: frontal; green: parietal; blue: temporal; magenta: occipital; yellow: insula; orange: cingulate; gray: inter‐lobar connections

**Figure 4:**
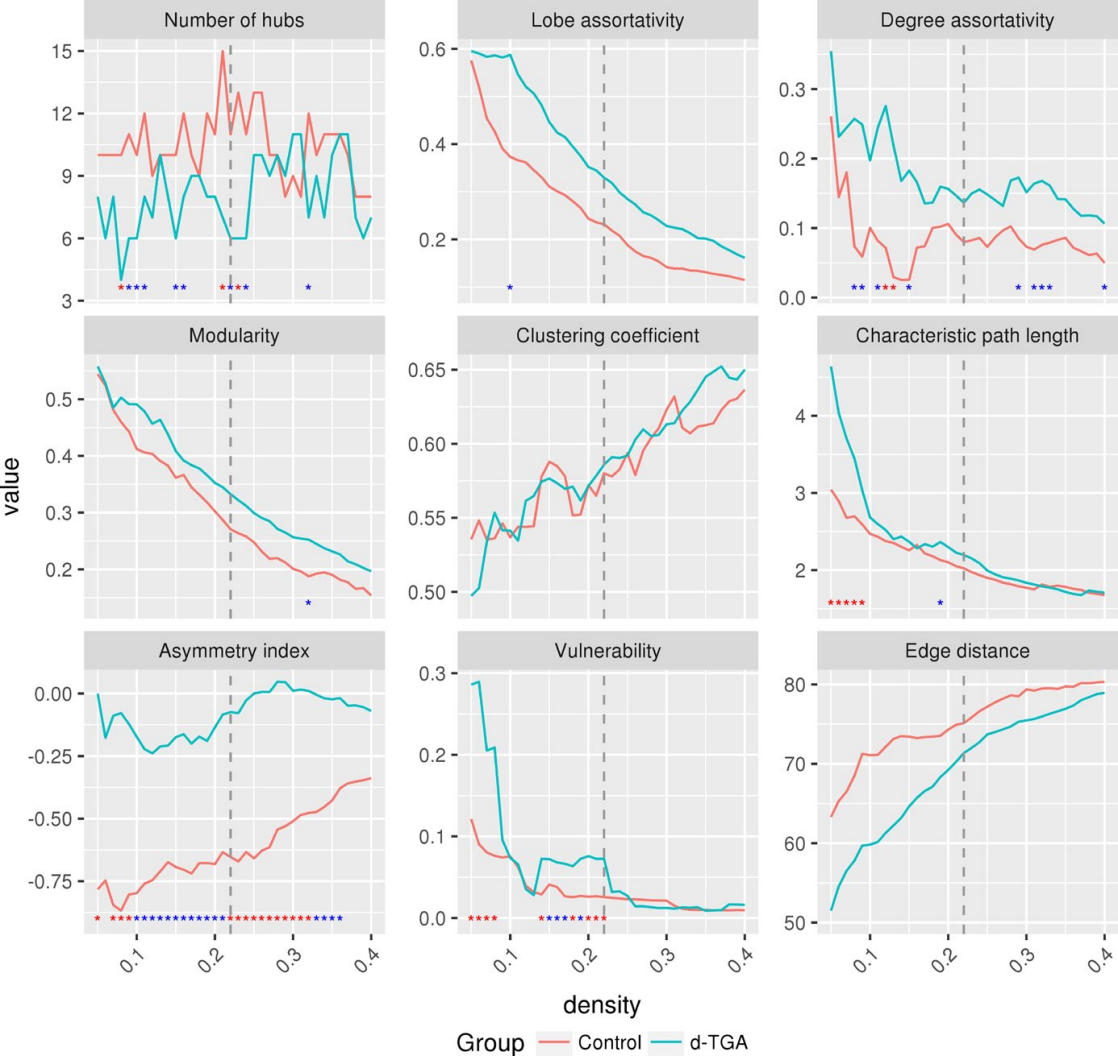
Global network measures plotted against density for the control and d‐TGA groups. Red asterisks indicate a significant (*p *< .05) group difference based on permutation testing (*N* = 10,000); blue asterisks indicate a trend (*p *< .1). The dashed vertical lines represent a density of 0.22

Figure 3 shows adjacency matrices for both groups at a density of 0.22 with vertices colored by lobe (inter‐lobar connections are colored gray). Qualitatively, the d‐TGA group has more fronto‐frontal and fronto‐parietal connections than the control group, and the control group has more fronto‐ and parieto‐temporal connections. This is reflected in the lobe assortativity, which was greater in the d‐TGA group at every network density tested, though the differences were not statistically significant (Figure 4). Additionally, both degree assortativity and modularity were consistently higher in the d‐TGA group, although the difference was not statistically significant across all densities (*p*_AUC_ = .08 and *p*_AUC_ = .28 for assortativity and modularity, respectively). Several other global network measures are also plotted against density in Figure 4.

**Figure 5:**
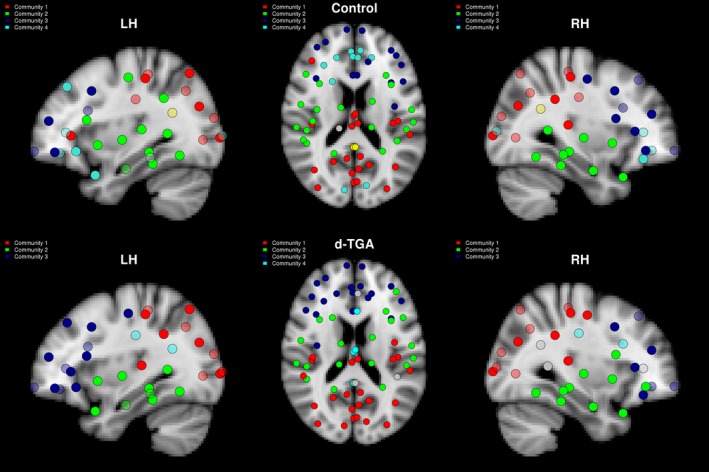
Modules in the control and d‐TGA groups at a density of 0.22. The control (top) and d‐TGA (bottom) groups have a similar distribution of modules. The left column depicts a sagittal view of the left hemisphere, the center column depicts and axial view, and the right column depicts a sagittal view of the right hemisphere. Colors of individual vertices are based on membership of specific module as detected by the Louvain algorithm — red: medial/posterior; green: temporal; blue: frontal; cyan: medial frontal/cingulate/occipital (top) and medial cingulate (bottom)

At a density of 0.22, both groups had 4 major (i.e., containing 3 or more vertices) modules detected by the Louvain algorithm (Figure 5): a bilateral medial/posterior module predominantly in occipital and parietal cortex; a bilateral module predominantly in temporal cortex; a bilateral medial module predominantly in frontal cortex; and a smaller module in bilateral cingulate cortex for the d‐TGA group, and a module in bilateral frontal/cingulate/occipital cortex in the control group.

### 3.6 Small‐worldness

**Figure 6:**
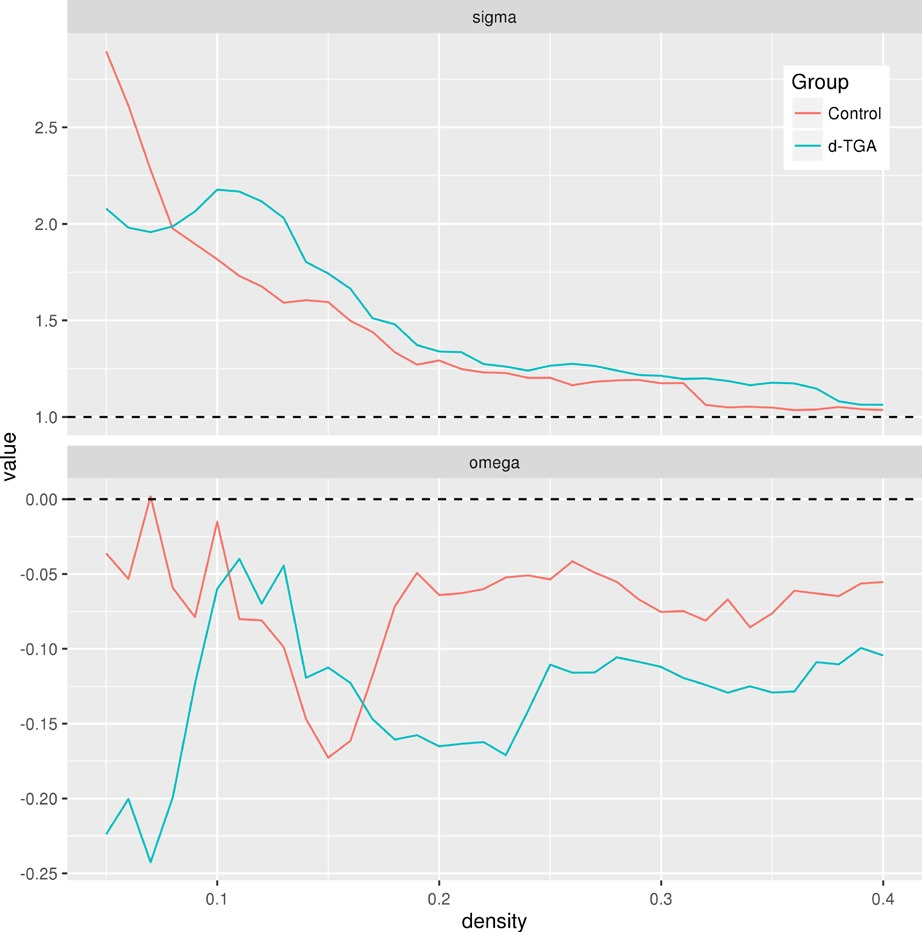
Small‐world coefficients plotted against density for the control and d‐TGA groups. The small‐world coefficients σ (top) and ω (bottom) are shown for the control (red) and d‐TGA (blue) groups. Top: The dashed line at *y* ****=** **1 indicates the minimum value for a network to be considered a small‐world network. Bottom: The dashed line at *y* ****=** **0 indicates the value at which a network is considered to display a balance between global clustering coefficient and characteristic path length

Figure 6 (top) shows the small‐world index, σ, plotted against network density for both groups. At all densities, σ > 1, indicating the presence of small‐world organization in both groups. In the bottom panel, an alternate small‐world index ω is plotted. For both groups, −0.25 < ω < 0, suggesting these networks possess the small‐world property and are closer to a lattice than a random network (Telesford et al., 2011).

### 3.7 Network closeness

**Figure 7:**
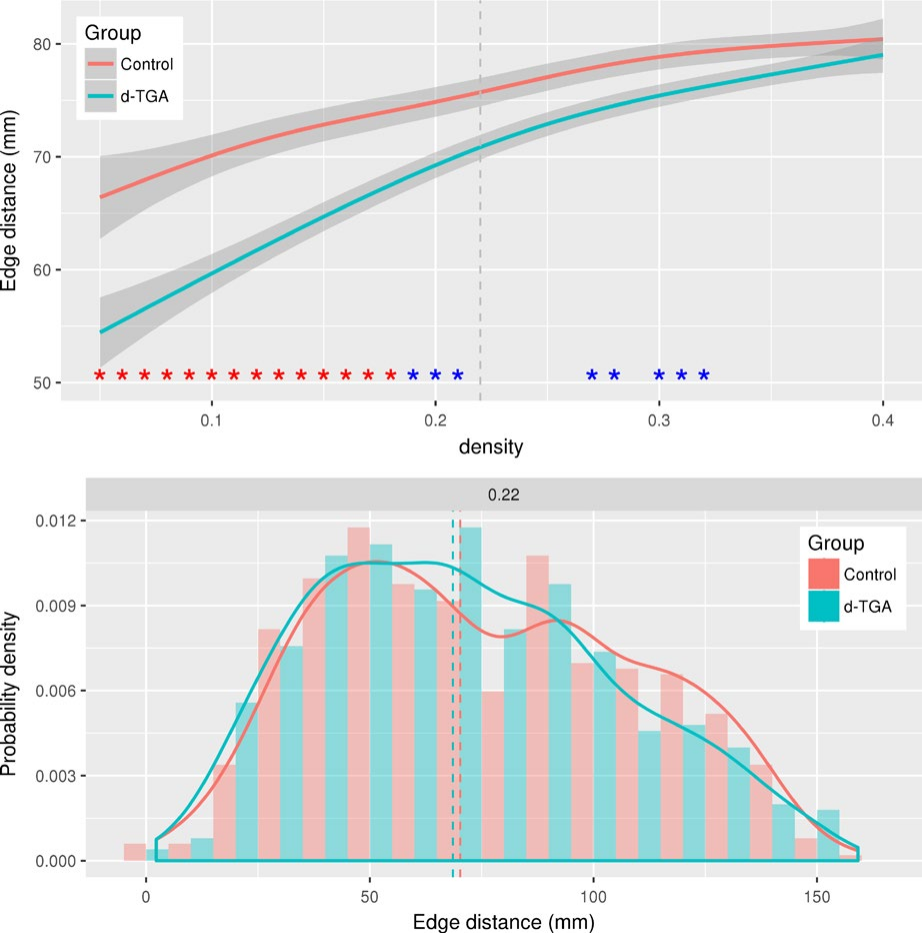
Edge distances. (Top) Median edge distances plotted against density for the control and d‐TGA groups. The shaded region indicates the 99% confidence interval across densities. Red asterisks indicate a significant (*p*_FDR_ < .05) group difference; blue asterisks indicate a trend (*p*_FDR_ < .1). The dashed vertical line represents a density of 0.22. (Bottom) Histogram of edge distances for the control and d‐TGA groups at a density of 0.22. The solid lines are group density curves, and the dashed vertical lines represent the group median edge distance

Characteristic path length was significantly lower in the control group across densities, indicating that regions are topologically closer to one another compared to the d‐TGA group (*p*_AUC_ = .026). Edge distance was significantly different between groups at several network densities (Figure 7, top), but not across all densities (*p*_AUC_ = .14). In all cases, the median edge distance was higher in the control group than in the d‐TGA group. Figure 7 (bottom) shows a histogram and density plot of edge distances for both groups at the network density of 0.22; while the between‐group difference was not statistically significant at this density (*p*_FDR_ = .11), the control group tended to have more long‐distance connections than the d‐TGA group.

### 3.8 Asymmetry and hemispheric efficiency

Asymmetry was negative and lower in the control group at all network densities (Figure 4), indicating a greater number of intrahemispheric connections in the right hemisphere than in the left. This between‐group difference was statistically significant at multiple network densities, including a density of 0.22 (*p* = .04). Across all densities, the between‐group difference did not reach statistical significance (*p*_AUC_ > .05). Additionally, a rightward asymmetry is evident in the control network's rich club, whereas the d‐TGA network has rich‐club regions distributed more evenly across hemispheres (Figure 2).

**Figure 8:**
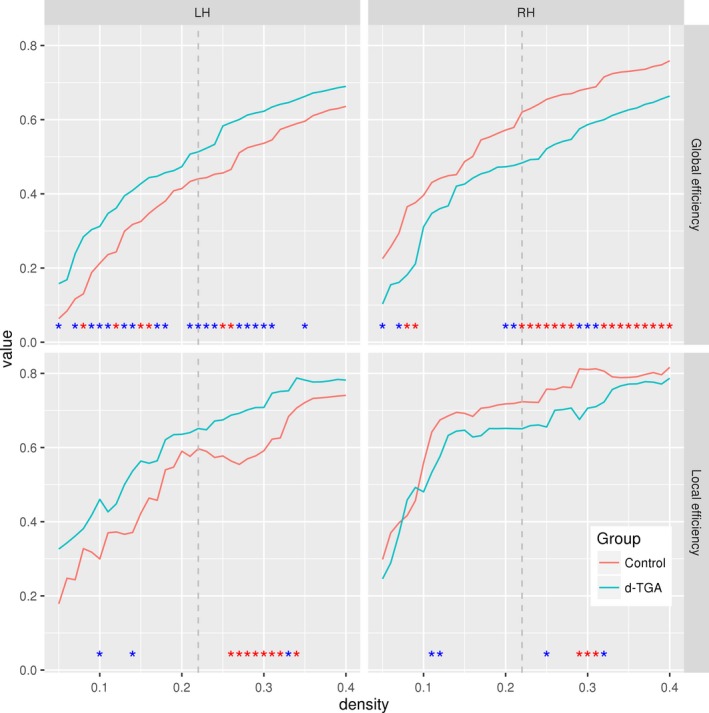
Global and local efficiency in individual hemispheres plotted against density for the control and d‐TGA groups. Red asterisks indicate a significant (*p* < .05) group difference based on permutation testing (*N* ****=** **1,000); blue asterisks indicate a trend (*p* < .1). LH and RH indicate left and right hemispheres, respectively

In the individual hemispheres, global efficiency was significantly higher in the control group than the d‐TGA group for the right hemisphere at multiple densities (statistically significant across all densities, *p*_AUC_ = .02) but was lower for the left hemisphere (statistically significant across all densities, *p*_AUC_ = .03) (Figure 8, top). In the control group, global efficiency was higher in the right than the left hemisphere at every density; in the d‐TGA group, global efficiency was lower in the right hemisphere or nearly equal to the left hemisphere. Local efficiency was higher in the left hemisphere for the d‐TGA group, but lower in the right hemisphere (except for two densities), compared to the control group (Figure 8, bottom). The between‐group difference in AUC of local efficiency was not significant for either hemisphere (*p*_AUC_ = .52 and *p*_AUC_ =* *.37 for the left and right hemispheres, respectively). In the control group, local efficiency was higher in the right hemisphere compared to the left at every density; in the d‐TGA group, there was no consistent pattern present.

### 3.9 Network robustness

**Figure 9:**
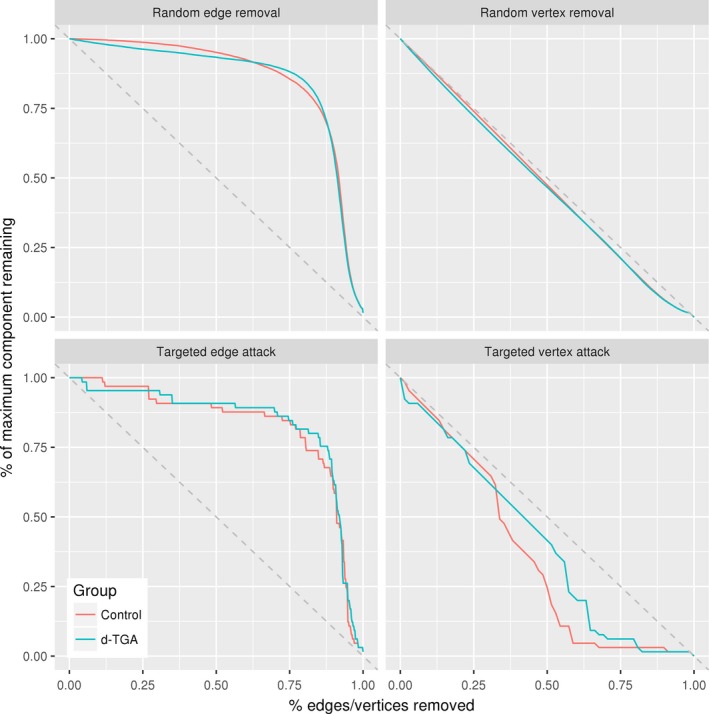
Robustness analyses for the control and d‐TGA groups at a density of 0.22. Relative maximal connected component sizes plotted as a function of: upper left, the percent of total edges removed in a random failure analysis; upper right, the percent of total vertices removed in a random failure analysis; lower left, the percent of total edges removed in a targeted attack analysis; and lower right, the percent of total vertices removed in a targeted attack analysis

At a density of 0.22, global vulnerability was significantly higher in the d‐TGA group compared to the controls (group difference = −0.049; *p* = .027); a similar relationship was seen at multiple densities (Figure 4). For the random failure and targeted attack analyses, at a density of 0.22, there were no significant differences between groups (Figure 9). Both groups' networks were resilient against random failure of vertices and edges, and were similarly resilient to targeted attacks of vertices and edges.

## 4 DISCUSSION

Using a graph theoretical approach to analyze cortical thickness networks, we found both qualitative and quantitative differences in brain organization between a group of adolescents with d‐TGA and healthy controls. The d‐TGA network tended to be more segregated, with higher modularity and assortativity. The control network had more long‐range connections and was significantly more asymmetric toward the right hemisphere, both in terms of number of connections and global and local efficiency. The control network also had more hub regions, which, in turn, demonstrated more long‐range connections distributed across both hemispheres and all the major lobes of the brain. Finally, global vulnerability was higher in the d‐TGA network for a range of network densities, indicating a lower resilience to vertex “attacks.” However, networks in both groups did display small‐world and rich‐club organization in addition to having similar modular structure. In summary, these results suggest that compromised brain networks in d‐TGA adolescents possess less efficient and less integrated information processing as compared to control adolescents.

Congenital heart disease affects brain structure and development as early as the 3^rd^ trimester in utero causing features of developmental immaturity (Clouchoux et al., 2013; Dimitropoulos et al., 2013; Licht et al., 2009; Limperopoulos et al., 2010; Miller et al., 2007). Both pre‐ and postoperatively, the most common location of brain injury involves white matter, sometimes manifested as periventricular leukomalacia (PVL) (Beca et al., 2013; Gaynor, 2004). White matter damage early in development can adversely affect neuronal number and organization in gray matter (Inder et al., 1999; Leviton & Gressens, 2007; Volpe, 2009). Interestingly, while neuroimaging and neuropathologic evaluation performed in the postoperative period in infants with CHD has yielded evidence of white matter injury, radiologic features of chronic white matter injury have not been evident on routine MRI performed in longer term follow‐up. However, in the current sample of d‐TGA and control adolescents, we have found distributed reductions in white matter FA in the d‐TGA group, in addition to altered global network organization based on white matter networks constructed from DTI tractography, despite the absence of gross white matter abnormalities (Panigrahy et al., 2015; Rivkin et al., 2013; Rollins et al., 2014). Importantly, in this same cohort of adolescents with d‐TGA we have found various alterations of cortical volume and thickness (Watson et al., 2016). The current analysis of cortical thickness networks extends our previous finding of altered white matter network segregation in d‐TGA adolescents to include alteration of gray matter networks, as well.

Graph theory analysis of gray matter networks has been successful in differentiating other patient groups from healthy controls. An analysis of adults with temporal lobe epilepsy showed similar global organization but altered hub distribution relative to healthy controls (Bernhardt et al., 2011). Similarly, in adolescents with scoliosis, both the patient and control networks showed small‐world organization, but an altered hub distribution; interestingly, there was an asymmetry in hub location matching the patient group's thoracic curves, suggesting a functional relevance of hub regions (Wang et al., 2013). In young adults with a history of childhood maltreatment, Teicher, Anderson, Ohashi, and Polcari (2014) found that regions involved in emotional processing possessed lower centrality in the proband group as compared to controls; the groups also had significantly different rich‐club organization (Teicher et al., 2014). Taken together, these findings indicate that analysis of cortical thickness networks can reveal large‐scale differences in connected brain regions known to underlie various disease states. Our findings of differences between d‐TGA adolescents and healthy controls are in accord with this literature.

In this study, hub regions for the control group were mostly right‐hemispheric and distributed across frontal, parietal, temporal, and occipital lobes. In contrast, hub regions for the d‐TGA group were more bilaterally distributed and tended to involve frontal, temporal, and cingulate regions only. A similar pattern of asymmetry was present in the rich clubs of both groups. In both groups, hubs were located in multi‐modal association areas, appropriate for their developmental stage (Khundrakpam et al., 2013). In the control group, there was strong right hemispheric occipito‐ and parieto‐frontal connectivity between hubs for most densities; these regions are broadly involved in attention and visuospatial functions. Altered asymmetry in cortical GM gyrification, particularly in frontal and temporal regions, has been found in HLHS fetuses (Clouchoux et al., 2013). The mechanisms contributing to such asymmetries remain unknown; however, the relatively reduced blood flow in the right internal carotid artery as compared to the left, may render cerebral tissue on that side more vulnerable to injury during periods of significant reduction in oxygen delivery in hypoxic or ishemic conditions (Bogren, Buonocore, & Gu, 1994). Finally, edge distances tended to be greater in the control group; long‐distance connections may provide “shortcuts,” and support improved integration among brain regions underlying different cognitive functions (Kaiser, 2011).

We, and others have reported that patients with d‐TGA and other CHD have a high incidence of attention deficit hyperactivity disorder (ADHD), as well as deficits in executive function, attention, and visuospatial functioning (Bellinger et al., 2003, 2011; Razzaghi, Oster, & Reefhuis, 2015; von Rhein et al., 2015). The right hemisphere's importance in attention has been established for several decades, particularly in the study of patients with spatial neglect (Brain, 1941; Corbetta & Shulman, 2002; Gainotti, Messerli, & Tissot, 1972; Mesulam, 1981). Unilateral neglect is more commonly the result of right‐hemisphere lesions and tends to be more severe compared to neglect consequent to left hemisphere lesions. In addition to study of stroke patients, abnormalities in right hemispheric structure and function have been found in children and adults with ADHD (Almeida et al., 2010; Epstein, Conners, Erhardt, March, & Swanson, 1997; Makris et al., 2007; Valera, Faraone, Murray, & Seidman, 2007; Vance et al., 2007). Network efficiency is representative of parallel information processing and fault tolerance (Latora & Marchiori, 2001). Importantly, right‐hemispheric asymmetry in efficiency has been shown to be present in healthy adult humans and non‐human primates, but deficient in schizophrenia patients (Iturria‐Medina et al., 2011; Sun, Chen, Collinson, Bezerianos, & Sim, 2017). The strong rightward asymmetry in the control group compared to the d‐TGA group is consistent with these findings and suggests that the differences in hemispheric connectivity and network efficiency of d‐TGA adolescents are associated with their long‐term neurodevelopmental challenges in the cognitive domains of executive function and attention.

Our study has limitations. Imaging data were acquired on two separate MRI scanners. However, the scanners were the same model (GE Twinspeed 1.5T), the image sequence was identical on both scanners, and both subject groups were balanced across scanning location. Additionally, we included a covariate for scanning location when performing linear models of cortical thickness. Second, there were more males in the d‐TGA group; we similarly adjusted for subject sex in the linear models. Finally, our patient group consists of patients with one type of CHD who underwent surgery at a single institution more than 20 years ago. Thus, our results may not be generalizable to patients with different CHD types.

## 5 CONCLUSIONS

Structural brain networks in adolescents with d‐TGA, as measured by inter‐regional cortical thickness correlations, differ in several aspects from a group of control subjects. Globally, both groups possessed small‐world architecture, but network segregation tended to be higher in the d‐TGA group. The control network was more asymmetric, containing more connections in the right hemisphere, and had a more efficient right hemisphere than the d‐TGA group. Additionally, the d‐TGA group had fewer long‐range connections. Locally, the d‐TGA group had fewer hub regions which were connected to closer brain regions than in the control group. Taken together, these differences in structural connectivity based on cortical thickness indicate differences in brain organization that likely relate not only to gray matter connectivity but also to underlying white matter differences we have seen in these patients. Further, these network differences constitute candidate measures to test for associations with the cognitive differences already identified between typically developing adolescents and those born with and treated for d‐TGA.

## CONFLICT OF INTEREST

There have been no identified potential conflicts of interest.
